# Iridoid glycoside Aucubin protects against nonylphenol-induced testicular damage in male rats via modulation of steroidogenic and apoptotic signaling

**DOI:** 10.1038/s41598-022-18148-1

**Published:** 2022-08-12

**Authors:** Israr UL Hassan, Nazia Ehsan, Muhammad Umar Ijaz, Tayyaba Afsar, Houda Amor, Ali Almajwal, Suhail Razak

**Affiliations:** 1 Department of Zoology, Wildlife and Fisheries, University of Agriculture, Faisalabad, Pakistan; 2 Department of Community Health Sciences, College of Applied Medical Sciences, King Saud University, Riyadh, Saudi Arabia; 3 Department of Obstetrics, Gynecology and Reproductive Medicine, Saarland University Clinic, Homburg, Germany

**Keywords:** Drug discovery, Plant sciences

## Abstract

Aucubin (AU) is one of the widespread compounds belonging to the group of iridoid glycosides, which possesses numerous beneficial properties. Nonylphenol (NP), is a synthetic environmental toxicant that has the potential to cause male infertility through excessive production of reactive oxygen species. In the current study, the remedial potential of Aucubin was assessed against NP-generated testicular damage in male rats. Animals were distributed into four groups and treated for 56 days in this study. Control-group (0.1% DMSO + food), NP group (100 µg/kg), NP + AU group (100 µg/kg + 5 mg/kg) and AU group (5 mg/kg). NP exposure significantly (*p* < 0.05) reduced the activity of antioxidant enzymes i.e., glutathione reductase, catalase (CAT), superoxide dismutase, glutathione peroxidase (GPx), and total protein content (TPC), whereas the level of reactive oxygen species (ROS) and thiobarbituric acid reactive substances (TBARS) was enhanced substantially (*p* < 0.05). Treatment with AU substantially (*p* < 0.05) recovered activities of antioxidant enzymes, TPC, ROS, and TBARS levels. Moreover, decrease in the levels of follicle-stimulating hormone (FSH), luteinizing hormone (LH), plasma testosterone, sperm count, motility, sperm membrane integrity, and the number of spermatocytes of different stages along with the level of steroidogenic enzymes i.e., 17β-hydroxysteroid dehydrogenase (17β-HSD), 3β-hydroxysteroid dehydrogenase (3β-HSD), and B-cell lymphoma 2 (Bcl-2) by NP administration were recovered to control values by AU treatment. However, AU mitigated the sperm abnormalities (head/midpiece/tail), the number of dead sperms, and proapoptotic proteins i.e., Bcl-2 associated X protein (Bax), caspase-9, and caspase-3 that were increased by NP. Besides, AU treatment recovered the NP-induced potential histopathological alterations in the testicular tissues such as the height of epithelium, seminiferous tubules diameter as well as the height of tunica propria. Overall, NP-induced toxicity was effectively recuperated by the AU administration. These results indicate that AU might be considered as a potential protective agent against testicular damage. The observed protection may be due to its antioxidant, anti-apoptotic, anti-inflammatory and androgenic potential.

## Introduction

Reproductive toxicity has resulted from the continuous liberation of a large amount of natural and man-made contaminants^1^ due to growing population, modernization, urbanization and industrialization^2^. Nonylphenol ethoxylates (NPEOs) are widely used in the preparation of detergents, plastics, resins, cosmetics, paints, paper, rubbers, textiles, herbicides and pesticides^3^. NPEOs degrades into nonylphenol (NP) by the action of microorganisms and ultraviolet light and subsequently it gets incorporated into the environmental matrices (water, soil, air, sediments and food) through anthropogenic activities^4^. Absorption, eye contact, skin contact, ingestion and inhalation are all possible routes of NP exposure. NP can accumulate and persist in the environment due to its hydrophobicity and long half-life, which may pose health hazards to animals and humans^5^. The concentration of NP in human blood samples varies widely, ranging from below the detection limit upto 53.21 ng/g^6^. Human urine samples were found to contain NP concentrations between 2.77 and 42.06 ng/mL^7^. In addition, studies conducted in Japan and Italy reported the presence of NP in human breast milk in the range of 0.65–1.4 ng/g^8^ and 13.4–56.3 ng/mL, respectively^9^. NP can cause testicular toxicity, neurotoxicity, liver damage and immunotoxicity^10^.

NP may act as an endocrine disruptor due to its structural similarity with estradiol, it mimics estrogen hormone, which can disrupt the reproductive system of organisms. NP can disrupt the body's pro-oxidant and antioxidant balance, resulting in oxidative stress (OS). This excessive ROS production can disturb the normal function of sperm i.e., acrosomal reaction, capacitation and hyperactivation^11^. OS may exert detrimental impacts on spermioteleosis, spermatogenesis, steroidogenesis and sperm qualities through lipid peroxidation, mitochondrial dysfunction, and apoptosis in the reproductive organs^12^. NP-generated OS is a key factor behind testicular dysfunction which may lead to male infertility. Furthermore, NP can cause cytotoxicity and apoptosis in Sertoli cells and Leydig cells that are pivotal to maintain testicular function^13^. Moreover, NP exposure significantly reduced the sperm motility, gene expression of testicular steroidogenic enzymes; steroidogenic acute regulatory protein (StAR), 3β-hydroxysteroid dehydrogenase (3β-HSD), 17β-hydroxysteroid dehydrogenase (17β-HSD), level of luteinizing hormone (LH), follicle-stimulating hormone (FSH), plasma testosterone and daily sperm production (DSP)^14^.

Medicinal plants produce various phytochemicals that are responsible for their preventive and healing properties. Iridoids are glycosides present in various plants and they are bound to glucose moieties. They have the general form of cyclopentopyran and they have molecular structure related to iridodial^15^. Iridoids are structurally classified into iridoid glycosides and non-glycosidic iridoids according to the presence or absence of intramolecular glycosidic bonds. AU chemically known as β-D-glucopyranoside is the natural iridoid glucoside present in the traditional Chinese plants *Eucommia ulmoides*, *Rehmannia glutinosa*, *Aucuba japonica* Thunb and *Plantago asiatica*^16^. Aucubin is reported to have several therapeutic potentials such as an anti-inflammatory^17^, antioxidant^18^, anti-aging^16^, anti-tumour^17^, anti-apoptotic^19^, hepatoprotective^20^ and nephroprotective effects^21^. Therefore, keeping in view the therapeutic role of AU, the present study was designed to explore the antioxidant potential of AU against NP-induced testicular toxicity by assessing antioxidant enzymes, lipid peroxidation, steroidogenesis, apoptosis, sperm profile, hormonal levels and histopathology.

## Materials and methods

### Animal ethics approval

All the experimental protocols for animal handling and treatment were reviewed and monitored by ethical committee of University of Agriculture, Faisalabad, in line with the European Union of animal care and experimentation (CEE Council 86/609) approved protocol. The study was carried out in accordance with ARRIVE guidelines.

### Chemicals used

Nonylphenol (IUPAC name: 4-(2,4-dimethylheptan-3-yl) phenol; CAS no: 84852-15-3; Purity: 99%) and Aucubin (IUPAC name: 5-Hydroxy-7-(hydroxymethyl)-1,4a,5,7a-tetrahydrocyclopenta[c]pyran-1-yl β-D-glucopyranoside: CAS no: 479-98-1; Purity: ≥ 98.0%) were purchased from Sigma Ald (Germany). All other chemicals used in the study were of analytical grade, such as dimethyl sulfoxide (DMSO), sodium pyrophosphate buffer, Na_3_PO_4_ buffer, phenazine methosulphate, glacial acetic acid, NADH, NADPH, sodium acetate buffer, N, N-diethyl-para-phenylenediamine, Ellman’s reagent, 5,5-dithiobisnitrobenzoic acid (DTNB), ascorbic acid, trichloroacetic acid and trichlorobarbituric acid were bought from Sigma Aldrich, Germany. Phosphate buffer saline (PBS) and H_2_O_2_ were purchased from Thermo Fisher, Germany.

### Experimental design and chemicals

Forty-eight male albino rats (190–210 g) were distributed into 4 equal groups (n = 12) at a standard temperature (25 ± 2 °C). Twelve hours of light and twelve-hour dark cycles were maintained. The control group was given only food and water by oral gavage. NP (100 µg/kg i.p.) was given to the NP-group, while the co-treated group (NP + AU) was provided with NP and AU (100 µg/kg + 5 mg/kg by i.p. respectively). AU only treated group was given AU (5 mg/kg i.p.). The 100 µg/kg dose of NP was used in accordance with a previous investigation conducted by Ganga et al.^22^, while a 5 mg/kg dose of AU was used in accordance to Xue et al.^23^. The whole experiment was conducted for 56 days. At the end of the experiment, rats were anesthetized by injecting ketamine/xylazine mixture via the intraperitoneal route^24^. Plasma was separated by collecting the trunk blood in heparinized syringes. Reproductive organs were removed. Blood was centrifuged for about 20 min at 322 × g and subsequently stored at − 20 °C for further assessment. After dissecting the rats, a 10% formalin buffer was used to fix the right testicle for histomorphometry and the left testis was preserved at −80 °C for biochemical analysis.

### Biochemical evaluation

#### Activity of catalase (CAT)

CAT activity was evaluated by following the protocol given by Aebi^25^. 50 μL tissue homogenate was diluted with 2 mL of phosphate buffer (7 pH). 2 mL diluted homogenate was mixed with phosphate buffer (1 mL) of pH 7 containing 30 mM of H_2_O_2_ in the test tube. Absorbance was noted at 240 nm by using spectrophotometer (UV–Visible/NIR-UH5700). CAT activity (1 unit) was expressed as unit mg^−1^ protein.

### Activity of superoxide dismutase (SOD)

The activity of SOD was assessed according to the procedure described by Kakkar et al.^26^. The reaction mixture was comprised of 1.2 mL of sodium pyrophosphate buffer (pH 7) and 0.1 mL of phenazine methosulphate. After centrifuging the 0.3 mL of supernatant (1500 × g for 10 min), the homogenate was poured into the reaction mixture. After that, 0.2 mL of NADH was added to initiate an enzymatic reaction, which was stopped by adding the 1 mL glacial acetic acid. With the help of a spectrophotometer, the chromogen amount was determined by recording absorbance at 560 nm by using spectrophotometer (UV–Visible/NIR-UH5700). The results were expressed in unit mg^−1^ protein.

### Activity of glutathione peroxidase (GPx)

GPx activity was evaluated according to protocol stated by Rotruck et al.^27^. The samples were incubated with hydrogen peroxide in the presence of glutathione for 10 min. The amount of used hydrogen peroxide was then ascertained by directly assessing GSH content using Ellman’s reagent, 5,5-dithiobisnitrobenzoic acid (DTNB) by using spectrophotometer (UV–Visible/NIR-UH5700). Its final values were exhibited as unit mg^−1^ protein.

### Activity of glutathione reductase (GSR)

GSR activity was calculated by estimating the NADPH disappearance following the methodology of Carlberg and Mannervik^28^. The change in absorbance was estimated at 340 nm by using spectrophotometer (UV–Visible/NIR-UH5700). NADPH was used as a substrate. An extinction coefficient of 6.22 × 10^3^ M^−1^ cm^−1^ was used for calculations. The values obtained were expressed as nM NADPH oxidized min^−1^ mg^−1^ tissue.

### Analysis of total protein (TPC)

The evaluation of TPC was carried out by the protein kit (Cat No. BR5202-S, AMEDA Labordiagnostik GmbH, Krenngasse, Graz, Austria). 20 µL of standard and supernatant was added to an Eppendorf tube containing 1 ml of reagent (R) and vortex to mix thoroughly. After 10 min of incubation at 37 °C optical density was measured against a reagent blank at 546 nm by using a spectrophotometer (UV–Visible/NIR-UH5700). Moreover, results were computed by plotting the absorbance of standard vs. sample absorbance on the graph. Final results were shown in mg g^−1^ of tissues.

### Thiobarbituric acid reactive substances (TBARS) level

The protocol developed by Wright et al.^29^ was used to estimate the TBARS level. Phosphate buffer (0.29 mL, pH 7.4), sample (0.1 mL), and 100 mM of ascorbic acid (0.1 mL) were mixed. Later on, incubation of the solution was carried out in a stirring water bath (at 37 °C) for about 1 h. 0.5 mL of trichloroacetic acid (10%) was added as a stop solution. After adding 0.67% of trichlorobarbituric acid (1 mL), test tubes were kept in a water bath (at 95 °C) for about 20 min. Later on, tubes were transferred to an ice bath and centrifuged at 2500 × g for 10 min. The quantity of TBARS was calculated by using a spectrophotometer (UV–Visible/NIR-UH5700) to measure supernatant optical density at 535 nm against a blank. Final data were noted as nM TBARS min^-1^ mL^-1^ tissue at 37 °C with the molar extinction coefficient of 1.56 × 10^5^ M^−1^ cm^−1^.

### Reactive oxygen species (ROS) level

ROS level was measured by following the procedure developed by Hayashi et al.^30^. Homogenate (5 μL) and 0.1 M sodium acetate buffer (140 μL) with pH 4.8 were mixed and dispensed in a 96-well plate. After incubating at 37 °C for 5 min, 100 μL of ferrous sulfate solution and N, N-diethyl-para-phenylenediamine were dispensed to each plate and then incubated at 37 °C for 1 min At 505 nm, the absorbance was observed with the help of a microplate reader (Mark™ microplate absorbance reader1681130) for 180 s with a 15-s interval. In the end, the standard curve was plotted. ROS was recorded as unit g^-1^ tissues.

### Estimation of sperm indices

#### Epididymal sperm count

A hemocytometer was used to count epididymal sperm, according to a procedure developed by Yokoi et al.^31^. The caudal region of the epididymis was minced using anatomical scissors in 5 mL of physiological saline solution, kept in a rocker for 10 min, and incubated at room temperature for about 3 min. The supernatant was diluted at 1:100 using a solution containing 1 mL formalin (35%), 5 g sodium bicarbonate, and 25 mg eosin per 100 mL of distilled water. A 10 μL drop of this mixture was placed in a sperm-counting chamber. Finally, at least 10 fields were observed under the light microscope (Nikon, 187,842, Japan) at 400x.

#### Sperm motility

Sperm motility was computed according to the method explained by Moumeni et al.^32^. The cauda epididymis was sliced and placed in 0.5 mL of prewarmed phosphate-buffered solution (pH 7.3) containing a drop of nigrosine stain. 50 μL of homogenate was kept in a pre-heated (35 °C) slide for observation under the light microscope (Nikon, 187,842, Japan) at 200X. 10 fields and 100 sperms per sample were observed. Semen samples were assessed in triplicate and then the mean of these 3 estimated values was considered as final sperm motility.

#### Sperm viability

Sperm viability was measured using eosin-nigrosine staining by following the method described by Halvaei et al.^33^. 25 μL of eosin-nigrosine stain was mixed with the semen sample. 15 mL of an aliquot from this sample was placed on the slide, and a smear was prepared and dried at room temperature. Finally, the slides were observed under the microscope at 40x. Unstained or white sperms were classified as alive, while (red) stained sperms were considered dead. About 300 spermatozoa were observed under the microscope (Nikon, 187,842, Japan), and the percentage of dead sperms was shown in percentage.

#### Sperm morphological abnormality

Sperm morphology was assessed using a Kwik-Diff™ staining kit (Thermo Scientific, Pittsburgh, PA, USA). Smears were prepared using 5 mL of the semen sample, then air-dried and stained with eosin-nigrosine. Finally, slides were rinsed with water to remove excess stain and observed under the light microscope at 400x. 300 random cells were carefully examined on all slides and head, mid-piece/neck, and tail abnormalities of sperms were assessed^34^.

#### Sperm membrane-integrity

The integrity of the sperm membrane was assessed by the hypo-osmotic swelling (HOS) test by following the procedure described by Correa and Zavos^35^. HOS test was carried out by placing 20 μL of semen in 180 μL of fructose solution keeping the osmotic pressure at 80 mOsm/L for about 20 min. After incubating and then mixing, the sperms were stained with eosin and nigrosine. Finally, 200 spermatozoa with swollen and non-swollen tails were counted using the light microscope (40 × magnification).

### Hormonal analysis

The levels of plasma testosterone (bio–Check Inc., USA Catalog No. BC-1115), FSH (Bio-Check Inc., USA Catalog No. BC-1029), and LH (bio–Check Inc., USA Catalog No. BC-1031) were assessed by enzyme-linked immunosorbent assay ELISA kits as per the guidelines of the manufacturer. 50 mL of assay diluent and 10 mL of plasma were added to a 96-well ELISA plate and incubation was performed for about 2 h at room temperature. Then, plates were rinsed with the deionized water and before adding 100 mL of peroxidase-conjugated immunoglobulin G (IgG) anti-FSH solution, anti-LH, or anti-testosterone in each well, incubation was carried out for maximum 2 h. Plates were again rinsed with deionized water, and substrate solution was added to wells and incubated for about 25 min at room temperature. 50 mL of stop solution was added to each well to terminate the reaction. Finally, the absorbance of FSH, LH, and plasma testosterone was recorded at 450 nm by using an ELISA plate reader (Mark™ microplate absorbance reader1681130). All samples were run in triplicates and conducted at the same time under the same conditions to avoid inter-assay variation.

### Evaluation of the steroidogenic enzymes and anti-or-apoptotic markers

The levels of 3β-HSD (Catalogue number-abx585472), 17β-HSD (Catalogue number-abx585472) Bcl-2 (Catalogue number-abx155246), Bax (Catalogue number- abx155246), caspase-3 (Catalogue number-abx155246), and caspase-9 (Catalogue number-abx155246) were assessed according to the guidelines provided by Abbexa kits, United Kingdom UK. 100 µL of each standard, control, and sample were added into the appropriate wells, which were covered and incubated for 1 h at 37 °C. In the next step, the cover was removed, and the liquid was discarded. Subsequently, 100 µL of the detection reagent A working solution was added to each well, and the plate was sealed with a cover and incubated for 1 h at 37 °C. Then the cover was removed and the solution was discarded. After that plate was washed three times with 1X Wash Buffer. In the next stage, 100 µL of detection reagent B working solution was added to each well, which was sealed and incubated at 37 °C for 30 min. Again, the solution was discarded, and the plate was washed five times with wash buffer as explained in the previous step. Likewise, a 90 µL aliquot of TMB Substrate was added to each well, and the plate was sealed with a cover and incubated at 37 °C for 10–20 min without exposure to light. Finally, 50 µL of Stop Solution was added to each well, and absorbance of 3β-HSD, 17β-HSD, Bcl-2, Bax, caspase-3, and caspase-9 were recorded at 450 nm immediately.

### Histopathological analysis

Samples of testicular tissues were preserved in 10% neutral phosphate-buffered formalin and dehydrated in ascending grades of alcohol. The 820-Spencer rotatory microtome was used to cut the 4–5 μm thick tissue segments and stained with hematoxylin–eosin stain. Ultimately, the segments were studied under the light microscope (Nikon, 187,842, Japan) at 40X, and images were taken by using a camera (Canon, EOS 250D). Images were examined by using Image-J2x software (https://sourceforge.net/projects/ij2x/).

### Statistical analysis

Tukey’s test was applied to compare various groups after values were subjected to one-way ANOVA for statistical analysis using GraphPad Prism 5 software (https://www.graphpad.com/support/prism-5). The results were displayed as Mean ± SD, and the significance level was considered as *p* < 0.05.

## Results

### Effect of NP and AU on the biochemical parameters

**Figure 1:**
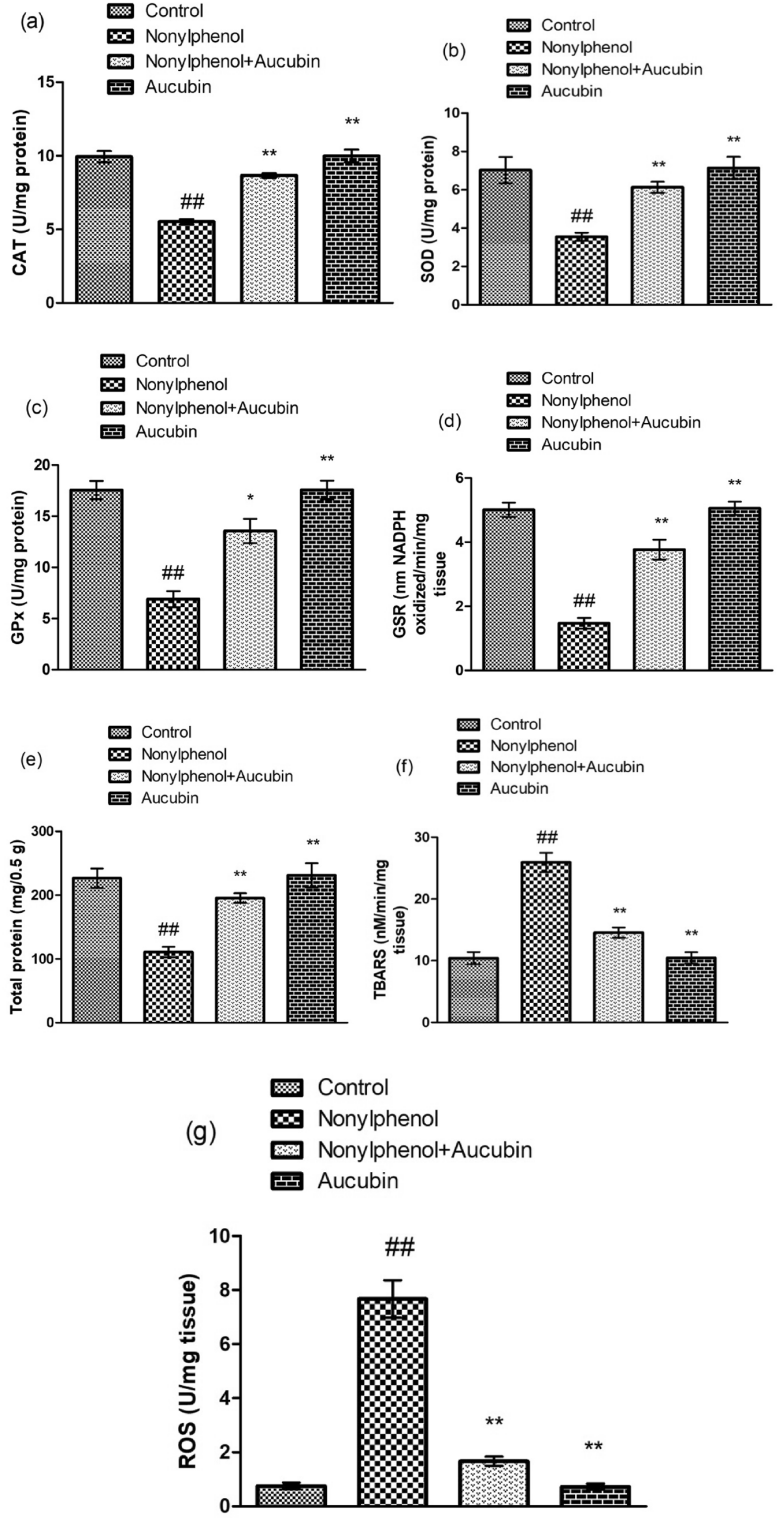
Effect of NP and AU on the biochemical profile in rat testicular tissues: catalase (CAT) (**a**), superoxide dismutase (SOD) (**b**), glutathione peroxidase (GPx) (**c**), glutathione reductase (GSR) (**d**), total protein content (TPC) (**e**), thiobarbituric acid reactive substances (TBARS) (**f**), and reactive oxygen species (ROS) (**g**). Values are expressed as Mean ± SD (12 rats per group). Significant differences displayed as ^##^ *P* < 0.01 compared to control; * *P* < 0.05, ** *P* < 0.05 compared to NP-treated group.

NP exposure significantly (*p* < 0.05) reduced the activities of antioxidant enzymes i.e., GSR, CAT, SOD, GPX, and TPC, while the levels of TBARS and ROS were reduced significantly (*p* < 0.05) compared to the control group. However, the co-administered group (NP + AU) displayed a significant (*p* < 0.05) upsurge in the antioxidant enzyme activities and TPC, while the TBARS and ROS levels were reduced compared to NP-treated groups. Moreover, the levels of TBARS, ROS, and the antioxidant activity of enzymes, as well as TPC in the AU alone, treated group was near to the control group (Fig. 1).

### Effect of NP and AU on the spermatogenic indices

**Figure 2:**
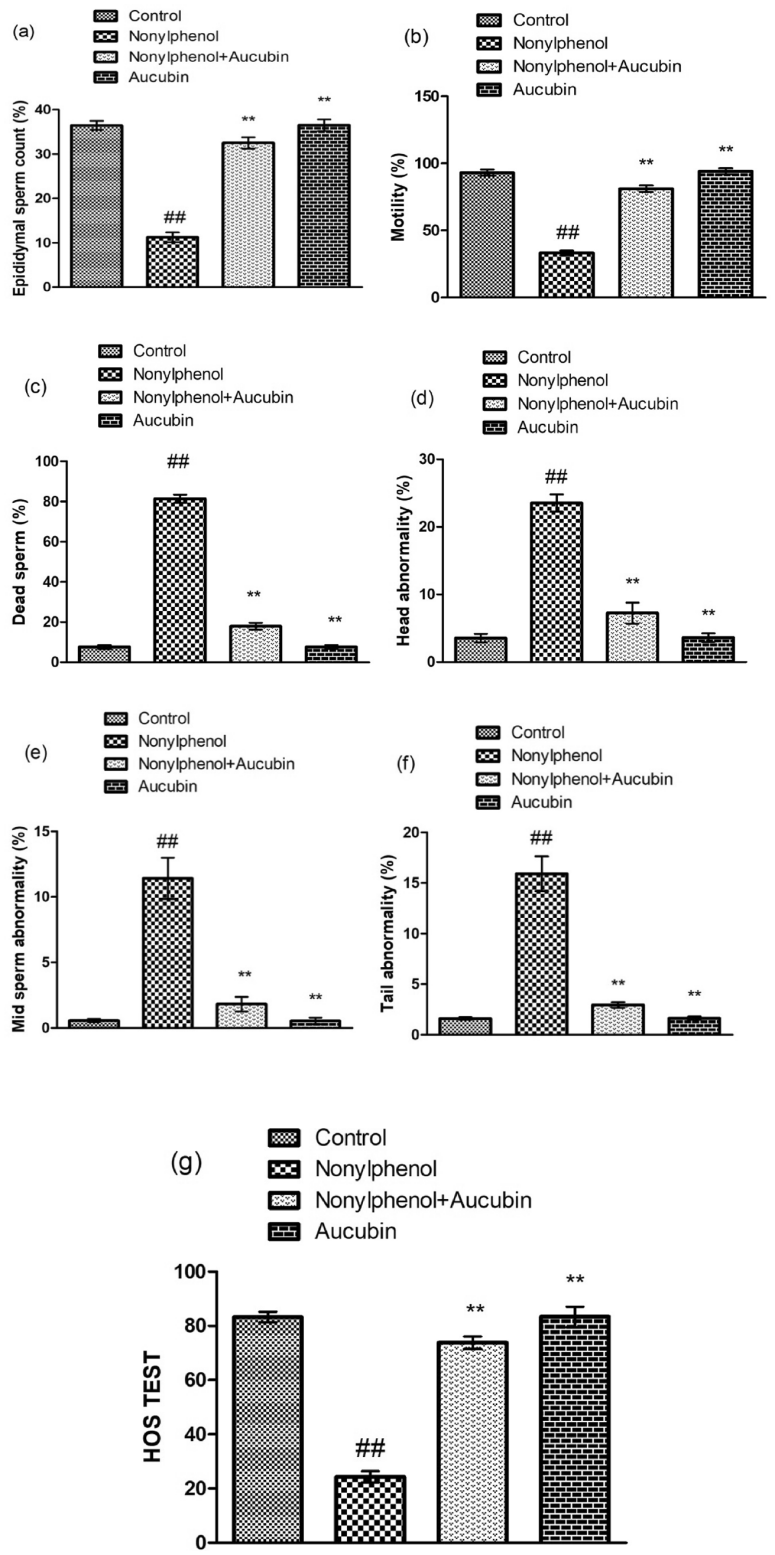
Effect of NP and AU on Spermatogenic-indices: epididymal sperm count (**a**), motility (**b**), dead sperm (**c**), head abnormality (**d**), mid sperm abnormality (**e**), tail abnormality (**f**), and sperm membrane integrity by hypo-osmotic swelling (HOS) test (**g**). Values are expressed as Mean ± SD (12 rats per group). Significant differences displayed as ^##^ *P* < 0.01 compared to control; **P < 0.05 compared to NP-treated group.

Nonylphenol exposure significantly (*p* < 0.05) decreased the percentage of spermatogenic indices such as sperm membrane integrity, motile sperm, and sperm count, while the percentage of sperm parts (tail, mid-piece, and head) and dead sperm was increased significantly (*p* < 0.05) as compared to control group. However, the co-administered group (NP + AU) exhibited a substantial (*p* < 0.05) upsurge in the spermatogenic indices such as sperm count, sperm motility, sperm membrane integrity, dead sperm, and sperm parts (head, mid-piece/neck, and tail) compared to NP-treated groups. Furthermore, the spermatogenic indices in the AU alone treated group was comparable to the control group (Fig. 2).

### Effect of NP and AU on the hormonal levels

**Figure 3:**
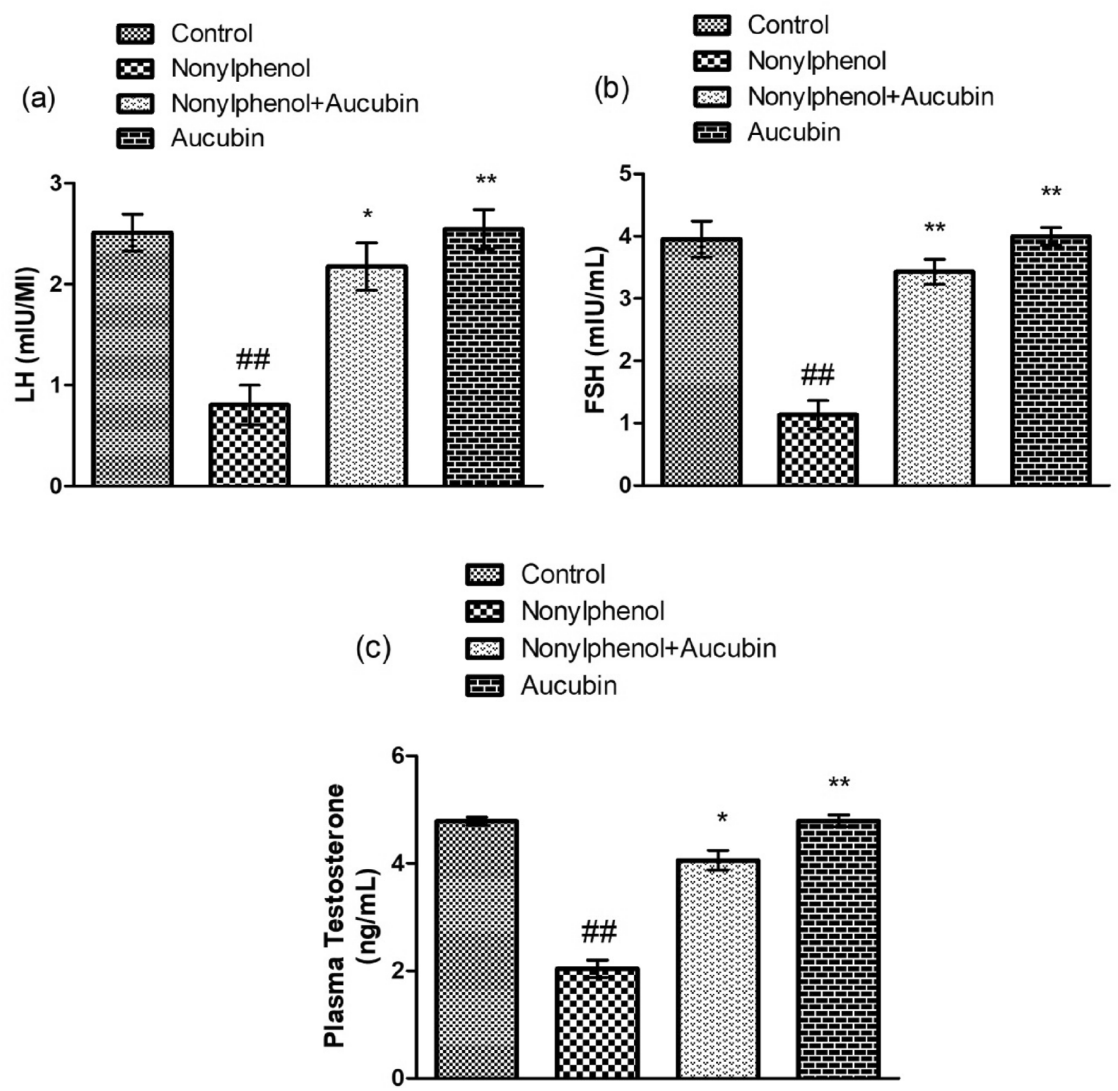
Effect of NP and AU on the levels of hormones: luteinizing hormone (LH) (**a**), follicle-stimulating hormone (FSH) (**b**), and plasma testosterone (**c**). Values are expressed as Mean ± SD (12 rats per group). Significant differences displayed as ^##^ *P* < 0.01 compared to control; *P < 0.05, **P < 0.05 compared to NP-treated group.

NP exposure significantly (*p* < 0.05) reduced the hormonal level such as LH, FSH, and plasma testosterone compared to the control group. However, the co-administered group (NP + AU) showed a substantial increase in concentrations of LH, FSH, and plasma testosterone compared to the control group. Moreover, hormonal levels of FSH, LH, and plasma testosterone in the AU alone treated group was comparable to the control group (Fig. 3).

### Effect of NP and AU on steroidogenic enzymes

**Figure 4:**
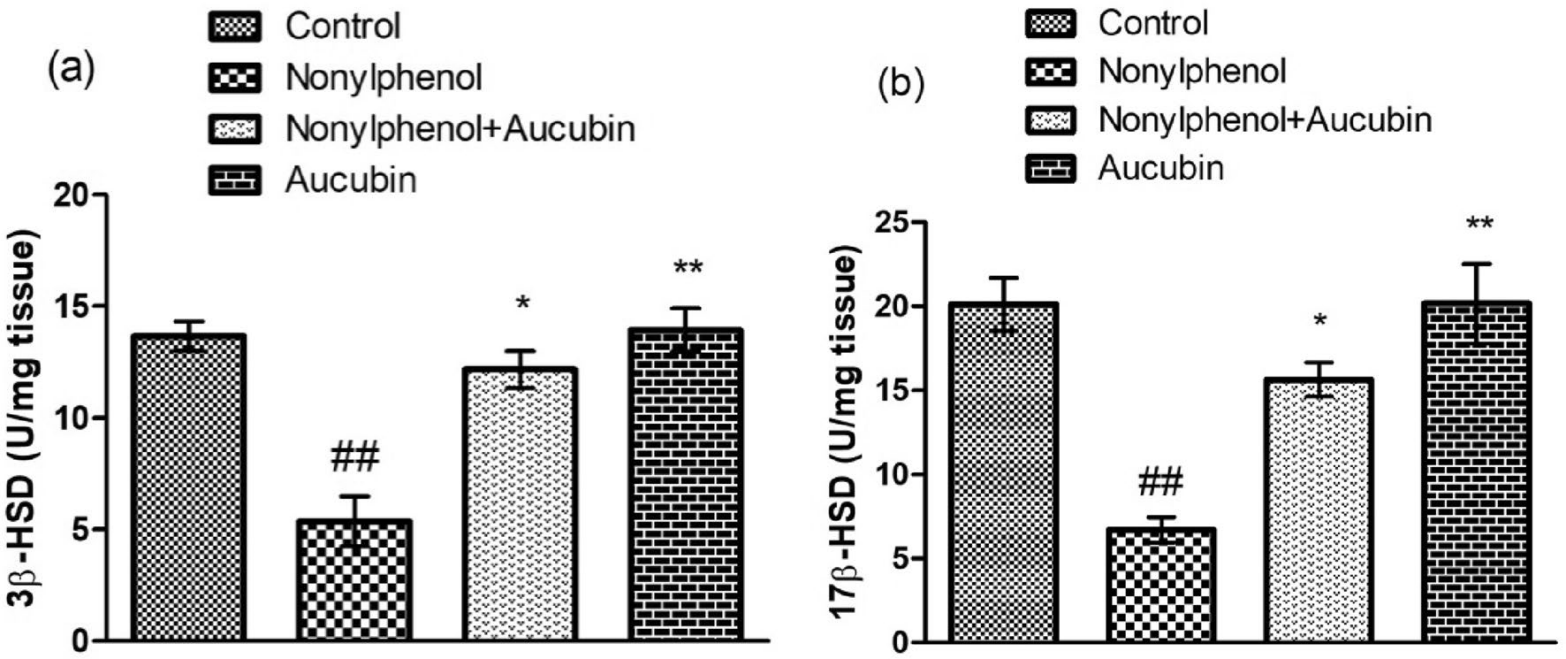
Effect of NP and AU on the 3β-hydroxysteroid dehydrogenase (3β-HSD) (**a**), and 17β-hydroxysteroid dehydrogenase (17β-HSD) (**b**) in rat testis. Values are expressed as Mean ± SD (12 rats per group). Significant differences displayed as ^##^ *P* < 0.01 compared to control; *P < 0.05, **P < 0.05 compared to NP-treated group.

NP treatment exhibited a significant (*p* < 0.05) reduction in the level of 3β-HSD and 17β-HSD as compared to the control group. However, the levels of 3-β-HSD and 17-β-HSD in the co-administered group (NP + AU) were increased as compared to NP group. Moreover, the levels of 17β-HSD and 3β-HSD in the AU alone treated group was close to the control group (Fig. 4).

### Effect of NP and AU on the apoptotic markers of testis

**Figure 5:**
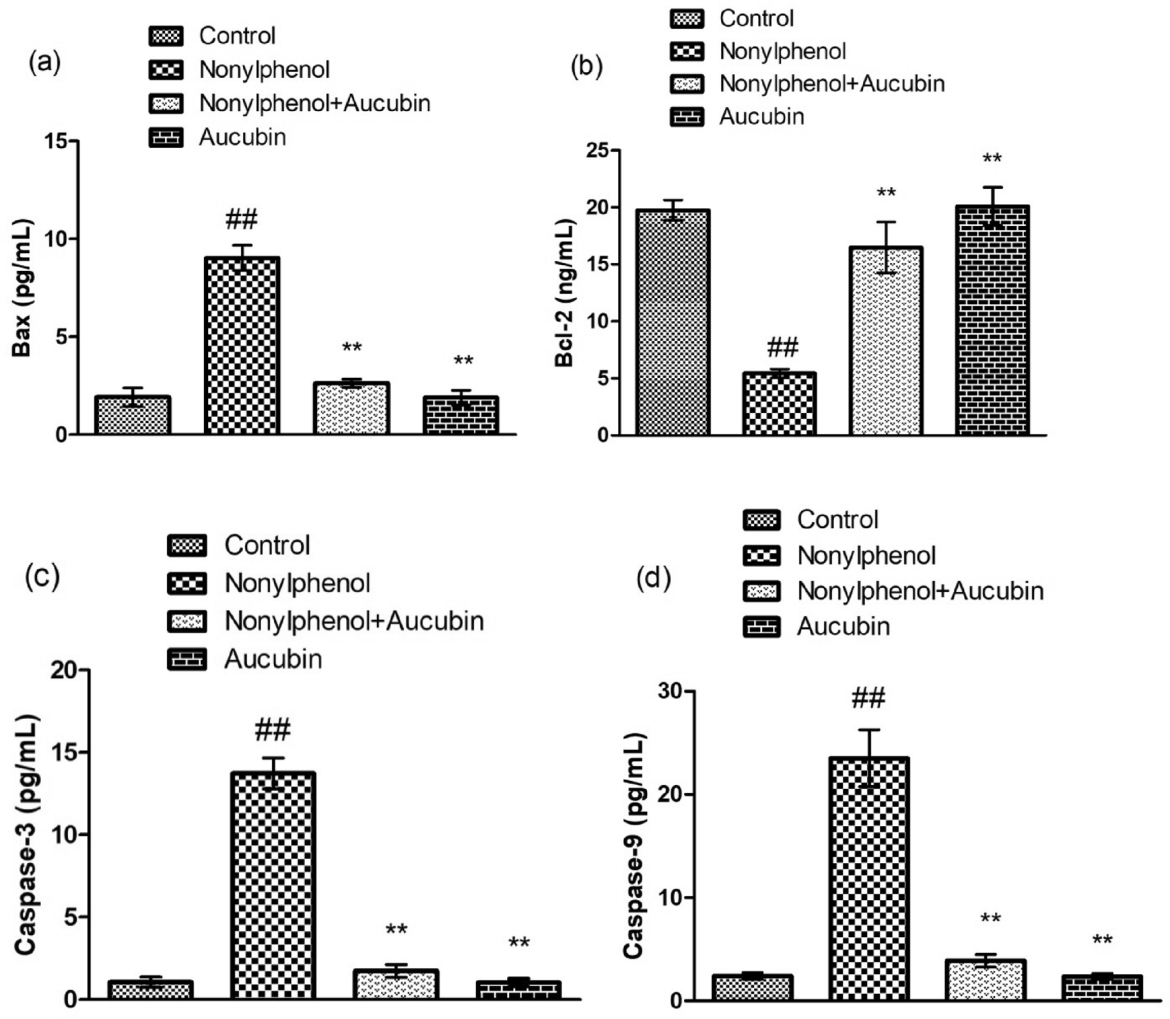
Effect of NP and AU on the testicular pro (Bax, Caspase-3, and Caspase-9)- or-anti-apoptotic (Bcl-2) markers. Values are expressed as Mean ± SD (12 rats per group). Significant differences displayed as ^##^ *P* < 0.01 compared to control; **P < 0.05 compared to NP-treated group.

NP-treatment significantly (*p* < 0.05) decreased the levels of Bcl-2, the levels of Bax, Caspase-3, and Caspase-9 were increased in contrast to the control group. However, the levels of Caspase-3, Caspase-9, and Bax were decreased whereas the Bcl-2 was increased in the Co-treated group (NP + AU). Moreover, the levels of these apoptotic markers in the AU alone treated group were close to the control group (Fig. 5).

### Effect of NP and AU on the histomorphology of testicular tissues

**Figure 6:**
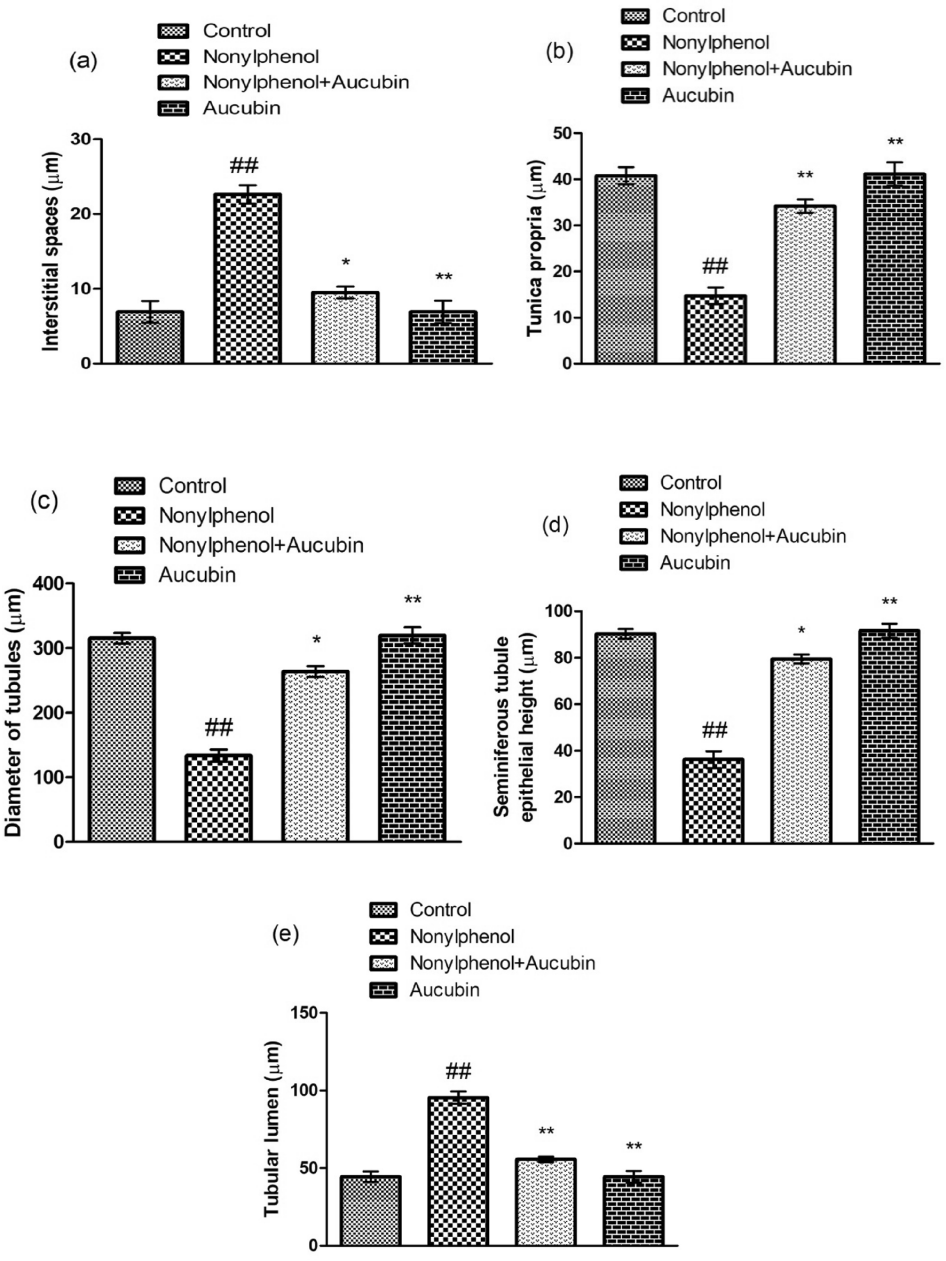
Effect of NP and AU on the testis interstitial spaces (**a**), tunica propria (**b**), diameter of tubules (**c**), seminiferous epithelial height (**d**), and tubular lumen (**e**). Values are expressed as Mean ± SD (12 rats per group). Significant differences displayed as ^##^ *P* < 0.01 compared to control; *P < 0.05, **P < 0.05 compared to NP-treated group.

**Figure 7:**
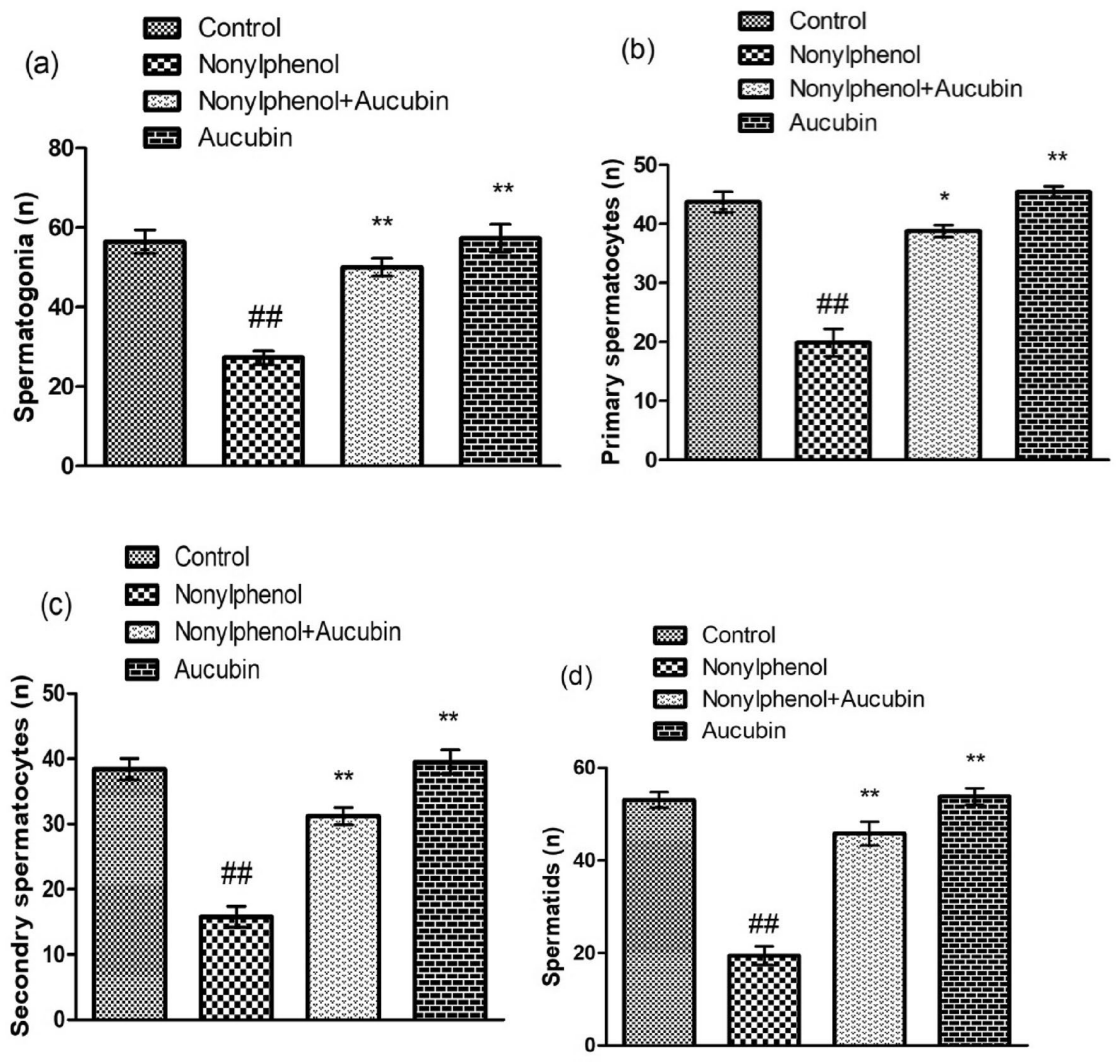
Effect of NP and AU on the spermatogonia (**a**), primary spermatocytes (**b**), secondary spermatocytes (**c**), and spermatids (**d**). Values are expressed as Mean ± SD (12 rats per group). Significant differences displayed as ^##^ *P* < 0.01 compared to control; *P < 0.05, **P < 0.05 compared to NP-treated group.

**Figure 8:**
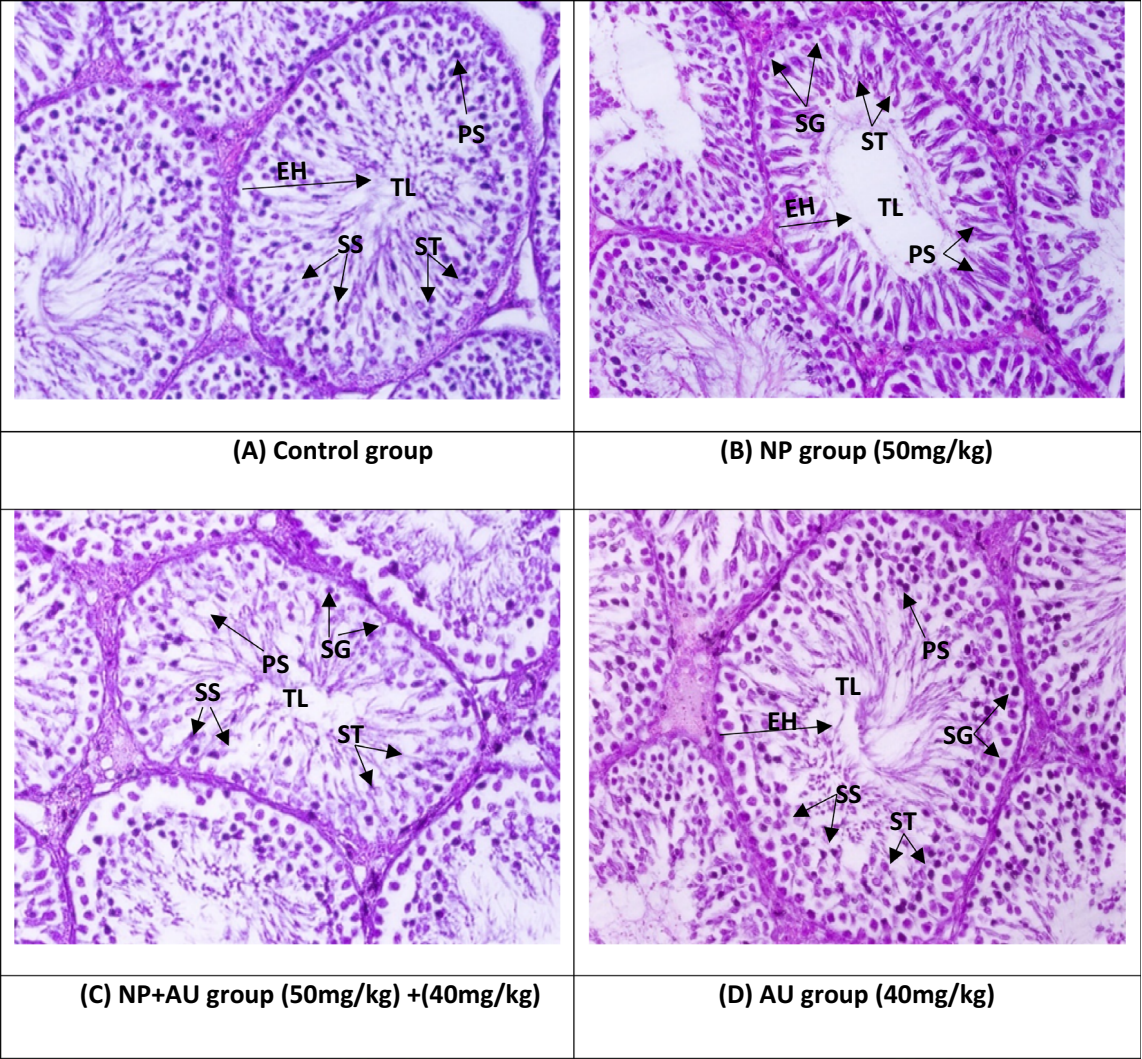
Shows histomorphometry examination of different groups of the testicular tissues (H & E, 400X) TP; Tunica propria, TL; Tubular lumen, IS; Interstitial spaces, EH; epithelium height, PS; Primary spermatocyte, SS; secondary spermatocyte, SG; Spermatogonia. A thick layer of epithelium and tunica propria along with normal interstitial spaces showed normal sperm production in the control group (**A**). However, NP-group (**B**) reveals a thin layer of epithelium, tunica propria with a large lumen and interstitial spaces showing damaged spermatocytes at different stages. NP + AU-Group (**C**) shows a prominent restoration in sperm production by improving the tunica propria, epithelium layer, interstitial spaces with tubular lumen, (**D**) the photomicrographs of control and AU group looked quite similar and no prominent changes were observed among them.

NP induced a significant (*p* < 0.05) decline in the seminiferous tubules’ diameter, height of epithelium along with the height of tunica propria (Figs. 6 and 8) and reduced the number of all spermatogenic stages (spermatogonia, primary spermatocyte, secondary spermatocyte, and spermatids) (Figs. 7 and 8), while significant (*p* < 0.05) enlargement in the tubular lumen and interstitial gaps were observed compared to the control group (Figs. 6 and 8). However, NP and AU co-treatment remarkably (*p* < 0.05) improved the morphometric deformities (Figs. 7 and 8) and increased the epithelium height along with the seminiferous tubule’s diameter and tunica propria width, whereas the interstitial spaces and tubular lumen were decreased in a co-administered group compared to the NP-group (Figs. 6 and 8).

## Discussion

Alkylphenol ethoxylates are being used globally in the polymer and chemical industry, 80% of it converts into NP thereby accumulating into the environment^4^. NP can deteriorate the testicular biochemical profile, steroidogenesis, hormonal balance and histological profile which may induce male reproductive dysfunction^36^. In this study, a plant-based antioxidant compound, Aucubin was used to alleviate the testicular damage induced by NP exposure. Iridoids are a class of active compounds that widely exist in the plant kingdom. In recent years, with advances in phytochemical research, many compounds with novel structures and outstanding activity have been identified^37^. Aucubin is a highly active compound possessing extensive biological effects including antioxidant, anti-aging, anti-inflammatory, anti-fibrotic, anti-cancer, hepatoprotective, neuroprotective, and osteoprotective properties^16^.

In this study, NP administration reduced the activities of antioxidant enzymes i.e., GSR, GPx, SOD, CAT, and TPC, while TBARS and ROS levels were increased in the NP-administered group. These antioxidant enzymes serve as the primary defense system against ROS that can damage lipids, proteins, and DNA. SOD transforms the superoxide anion to hydrogen peroxide while GSR provides reduced GSH for the continuous antioxidant activity of GPx. CAT acts on H_2_O_2_ converting it into H_2_O and O_2_ molecules. GPx transforms the lipid peroxide to lipid alcohol and H_2_O_2_ to H_2_O. Increased lipid peroxide levels have a direct impact on membrane fluidity and integrity, resulting in altered membrane protein function^38^. Shalini et al. reported that normal GPx activity is essential for male fertility and spermatogenesis. During normal cellular metabolism, free radicals such as hydrogen peroxide (H_2_O_2_), superoxide anion(O^−2^) hydroxyl-radical (OH), and nitric oxide (NO) are produced^39^. A high level of these free radicals is toxic to cells, tissues, and organs while the endogenous antioxidant enzymes protect them from damage^40^. Nevertheless, these detrimental changes were effectively mitigated by AU treatment, presumably because of its antioxidant activity. Our results are in line with the study conducted by Wu et al. who reported that AU treatment increased the activity of antioxidant enzymes and decreased the oxidative stress in cardiac tissues of rats^41^.

NP treatment remarkably decreased the ratio of sperm count, sperm motility, and sperm membrane integrity whereas it increased the percentage of dead sperm, head, mid-piece, and tail abnormality of sperm. OS is one of the major factors that adversely affect sperm structure, sperm motility, sperm membrane integrity, and viability^42^. During spermatogenesis, the antioxidant capacity of spermatozoa decreases therefore NP-generated ROS can easily attack spermatozoa’s mitochondrial membrane containing a large amount of polyunsaturated fatty acid molecules^40^. It causes lipid peroxidation of the sperm mitochondrial membrane resulting in low production of ATP. Low production of ATP affects the function of sperm flagella which ultimately affects sperm motility^43^. Overall, excessive production of ROS causes a detrimental impact on the spermatocytes which may lead to reduced sperm membrane integrity, viability, and motility and damaged the structure of sperm (head, mid-piece, and tail)^44^. However, AU treatment recovered the above-stated spermatological impairments.

In the present study, NP-treatment reduced the levels of FSH, LH, and testosterone. FSH can act individually or along with testosterone to instigate the Sertoli cells for nutrients and signaling to assist in Spermioteleosis^45^. Whereas, LH stimulates the Leydig cells to yield testosterone for spermatogenesis^46^. Therefore, FSH, LH, and testosterone are assumed to be required precisely for normal spermatogenesis and Spermioteleosis^47^. Leydig cells injury caused by the generation of OS in the testis leads to the reduced production of testosterone. This low production of testosterone leads to low sperm count^48^. These alterations are attributed to OS or derangement in the hypothalamic-pituitary–gonadal (HPG) axis and its communication with other endocrine glands, which may result in testicular dysfunction ^49^. However, AU treatment normalized NP-induced hormonal disturbance which may be due to safeguarding the HPG axis from the harmful effects of NP.

In the present study, the levels of steroidogenic enzymes were assessed to explore the rationale behind the low level of testosterone. Key androgenic enzymes (17β-HSD and 3β-HSD) catalyze the cholesterol into testosterone. In the current study, NP exposure substantially suppressed the levels of the 17β-HSD and 3β-HSD (steroidogenic enzymes) which in turn decreased the testosterone production in the testis^50^. Moreover, suppression of these key androgenic enzymes may be associated with the disturbance in the HPG axis^51^. However, AU administration remarkably increased the level of the abovementioned enzymes, which may be attributed to the androgenic nature of AU.

NP treatment remarkably increased the levels of apoptotic markers i.e., Bax, caspase-3, and caspase-9 whereas the levels of Bcl-2 protein (anti-apoptotic) were decreased. Caspase-3 protein performs one of the crucial roles during apoptosis. OS encourages the apoptotic process via caspase-3 protein stimulation in the body tissues which in turn causes apoptotic damage. Furthermore, Bax, Caspase-3, and Caspase-9 are pro-apoptotic proteins whereas Bcl-2 is an anti-apoptotic protein^52^. Reduced Bcl-2 levels and increased Bax levels promote cytochrome c to move from the mitochondrial membrane to the cytoplasmic matrix, which activates the caspase-9 and caspase-3 proteins, resulting in apoptotic damages^53^. However, AU treatment mitigated all the damages by increasing the Bcl-2 level while decreasing the level of caspase-3, Bax, and caspase-9 owing to its anti-apoptotic nature^19^.

In the current study, NP exposure induced a notable reduction in the seminiferous tubules’ diameter, and height of epithelium with the height of tunica propria and damaged all stages of spermatocytes while a remarkable (*p* < 0.05) increment in the tubular lumen and interstitial spaces were observed. These morphological alterations in testicular tissues caused depletion in the number of germ cells due to excessive production of ROS after NP exposure. The decrease in testosterone production eventually disturbed spermatogenesis^54^. However, AU has the potential to protect the blood-testicular-barrier^55^, and this may account for its histo-protective effects on testes, as evident from the outcomes of the present investigation in which AU treatment notably restored the histopathological changes as well as increased the number of germ cells belonging to different stages.

## Conclusion

Taken together, NP treatment deteriorated the endogenous antioxidant activities, steroidogenic enzymes, hormonal level, apoptotic markers, and sperm profile resulting in reproductive impairment. NP exposure disrupted the antioxidant system and subsequently resulted in excessive ROS production in the rat testis, which eventually led to testicular damage. Overall, these pernicious effects were recovered by AU exposure, probably because of its antioxidant and androgenic potential.

## Data Availability

All the data is contained in the manuscript.

## Abbreviations

AU: Aucubin
NP: Nonylphenol
GPx: Glutathione peroxidase
CAT: Catalase
T: Plasma testosterone
FSH: Follicle-stimulating hormone
GSR: Glutathione reductase
ROS: Reactive oxygen species
Bcl-2: B-cell lymphoma-2
LH: Luteinizing hormone
13-βHSD: 13-β-Hydroxysteroid dehydrogenase
17-β-HSD: 17-β-Hydroxysteroid dehydrogenase
SOD: Superoxide dismutase
OS: Oxidative stress
Bax: Bcl-2 associated x protein
TP: Tunica propria
TBARS: Thiobarbituric acids reactive substances
